# Increased Vertebral Gallium-68-DOTATATE Activity on Positron Emission Tomography-Computed Tomography in Merkel Cell Carcinoma: Not Always Metastasis

**DOI:** 10.7759/cureus.39427

**Published:** 2023-05-24

**Authors:** Kush Purohit, Greg Minassian, Luv Purohit, Robert Peyster, Avraham Bluestone

**Affiliations:** 1 Radiology, Stony Brook University Hospital, Stony Brook, USA; 2 Internal Medicine, Broward Health Medical Center, Fort Lauderdale, USA

**Keywords:** nonmelanoma skin cancer, pet-ct, metastasis, hemangioma, gallium-68-dotatate, merkel cell carcinoma

## Abstract

Merkel cell carcinoma (MCC) is a rare neuroendocrine dermal malignancy seen in elderly light-skinned individuals, associated with immunosuppression and Merkel cell polyomavirus infection. As a neuroendocrine tumor, the recurrence and metastasis of MCC can be evaluated using positron emission tomography-computed tomography (PET-CT) with the Gallium-68-DOTATATE (Ga-68-DOTATATE) radiotracer, which has demonstrated increased sensitivity to neuroendocrine metastases when compared to F-18 fluorodeoxyglucose (FDG). Here, we present the case of a patient with known metastatic MCC with a new, abnormal focus of increased radiotracer activity in the thoracic spine on Ga-68-DOTATATE PET-CT suspected to represent a metastatic lesion. Further evaluation with MRI revealed a benign vertebral hemangioma, highlighting the limitations of this radiotracer in the setting of benign spinal lesions. Multimodality imaging findings of metastatic MCC and potential pitfalls of Ga-68-DOTATATE PET-CT staging are discussed.

## Introduction

Merkel cell carcinoma (MCC) is a rare, deadly neuroendocrine tumor of the dermis, with 95% of cases reported in the elderly Caucasian population [1-3]. Additional risk factors for the disease include immunosuppression and Merkel cell polyomavirus infection, the latter of which has been reported in the vast majority of cases in the United States and Europe [4].

As with most dermatologic malignancies, a comprehensive physical examination with a biopsy of the lesions typically yields samples for definitive histopathologic diagnosis. Radiologic imaging may be performed in atypical cases for cancer staging and to monitor for recurrence. A variety of radiotracers have been developed for the imaging of neuroendocrine tumors using positron emission tomography-computed tomography (PET-CT), including F-18 fluorodeoxyglucose (FDG), Gallium-68-DOTATATE (Ga-68-DOTATATE), and Gallium-68-pentixiafor (Ga-68-pentixiafor), with varying sensitivity and specificity [5,6].

The case presented here uniquely highlights the use of Ga-68-DOTATATE in a patient with known metastatic MCC to illustrate the clinical utility of this radiotracer, as well as the potential pitfalls during evaluation for disease recurrence. Multimodality imaging findings are discussed.

## Case presentation

Initial presentation

An 84-year-old Peruvian male with a history of hypertension, coronary artery disease, and atrial fibrillation presented for the evaluation of an enlarging lump on the posterior left upper arm. He initially noticed a painless, erythematous nodule which rapidly increased in size over a period of two months while out of the country. The patient also reported feeling a new lump in the left axilla. On physical examination, the patient’s vital signs were within normal limits. A large erythematous mass was noted at the posteromedial aspect of the left upper extremity, overlying the triceps. An additional palpable mass was identified in the left axilla. No cervical or supraclavicular lymphadenopathy was present.

Initial imaging

**Figure 1:**
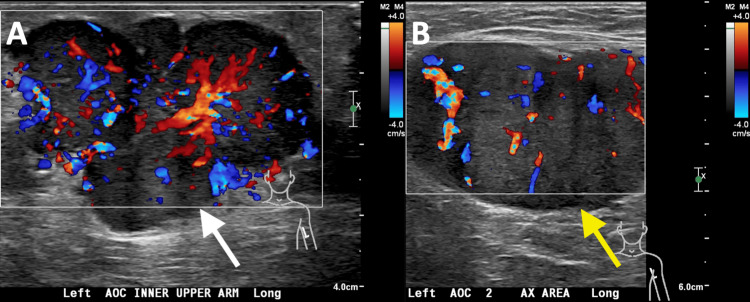
A. Targeted ultrasound of the left upper arm with color Doppler demonstrating a circumscribed, hypoechoic, polypoid mass (white arrow) with increased internal vascularity. B. Targeted ultrasound of the left axilla revealing a well-circumscribed, hypoechoic oval mass (yellow arrow) with increased internal vascularity.

Targeted ultrasound of the left upper extremity and axilla with color Doppler was performed. The study revealed an irregularly marginated, lobulated solid hypoechoic mass measuring up to 5.2 cm in the superficial soft tissues of the left upper arm with increased color Doppler flow (Figure 1A), raising suspicion for a neoplastic process. An additional solid hypoechoic mass measuring up to 7.4 cm with increased internal vascularity was identified in the left axilla (Figure 1B), which was suspected to represent an enlarged lymph node.

**Figure 2:**
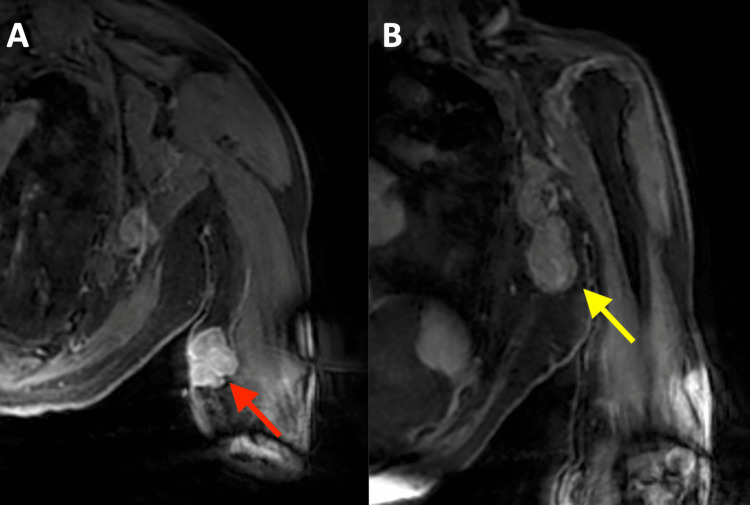
A. Coronal image of a post-contrast T1-weighted MRI sequence with fat suppression of the left upper extremity revealing an avidly enhancing polypoid mass (red arrow) within the soft tissues overlying the left triceps musculature. B. Additional coronal image of a post-contrast T1-weighted MRI sequence with fat suppression demonstrating multiple enlarged and enhancing left axillary lymph nodes (yellow arrow). MRI: magnetic resonance imaging

A magnetic resonance imaging (MRI) study with a gadolinium-based contrast agent of the left upper extremity and axilla was then performed. Coronal post-contrast T1-weighted imaging with fat suppression of the left upper extremity (Figure 2A) demonstrated an avidly enhancing polypoid subcutaneous mass overlying the left triceps musculature. Additional coronal images of the left axilla (Figure 2B) revealed bulky masses in the left axillary lymph node chain, consistent with lymphadenopathy, as suspected on the prior ultrasound.

Biopsy and pathology

Ultrasound-guided core needle biopsy of the left upper extremity and axillary masses was performed. Histopathologic analysis of the sample tissues revealed a round cell tumor with focal necrosis and high nuclear/cytoplasmic ratio staining positive for CK20, chromogranin, and synaptophysin, consistent with Merkel cell carcinoma.

Initial staging and treatment

**Figure 3:**
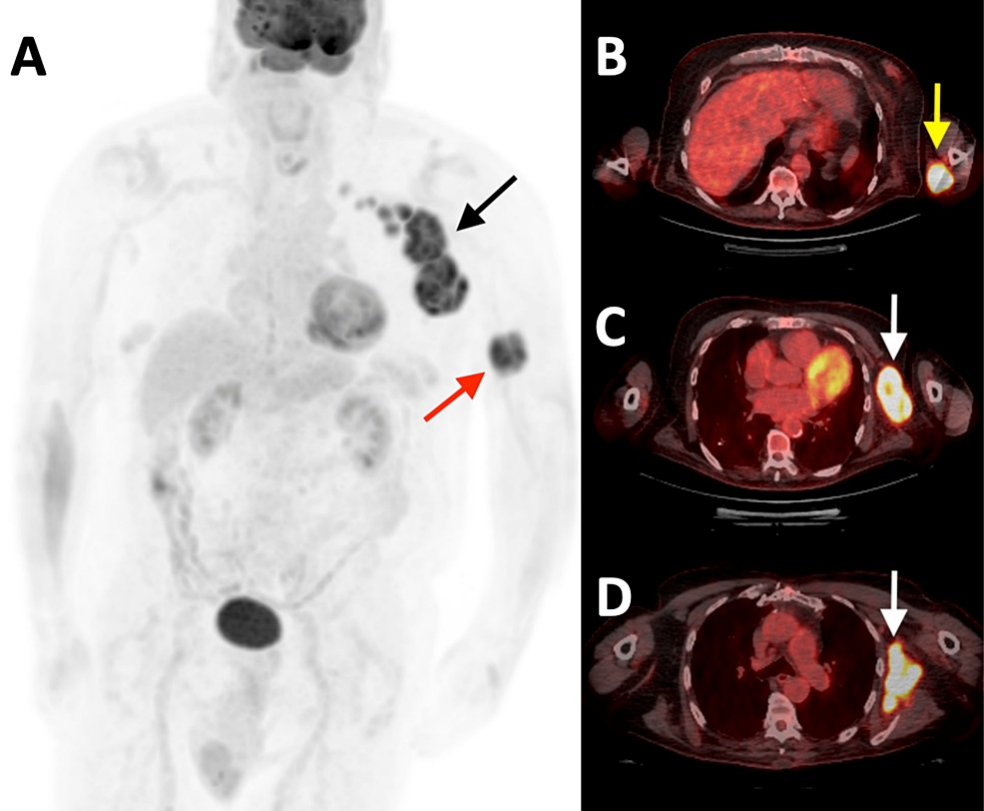
Pre-treatment Ga-68-DOTATATE PET-CT study. A. Coronal maximum intensity projection image of a Ga-68-DOTATATE PET study illustrating focally increased radiotracer uptake in the left upper arm (red arrow), consistent with a neuroendocrine tumor. Increased radiotracer uptake is also demonstrated in the left axilla (black arrow) with multiple discrete enlarged axillary lymph nodes. B-D. Axial fused images of a PET-CT examination with Ga-68-DOTATATE revealing focally increased radiotracer uptake within a left posteromedial upper arm mass (yellow arrow) with increased radiotracer activity corresponding to bulky left axillary lymphadenopathy (white arrows). PET-CT: positron emission tomography-computed tomography

A Ga-68-DOTATATE PET-CT was subsequently performed for staging purposes. Increased radiotracer uptake was demonstrated in the left upper extremity and axilla on the maximum intensity projection (MIP) image (Figure 3A), corresponding to the known masses seen in prior studies. Increased radiotracer activity was noted in the primary lesion overlying the triceps (Figure 3B) and in several enlarged axillary lymph nodes (Figures 3C, 3D). No additional foci of increased radiotracer uptake were identified.

Given the extent of left axillary lymph node spread, the patient was treated with four cycles of intravenous carboplatin (dose adjusted for AUC 5), etoposide 100 mg/m^2^, and atezolizumab 1,200 mg/20 mL chemotherapy and immunotherapy. This was followed by targeted external-beam X-ray radiation therapy to the left upper arm and axillary region with 25 fractions of 200 cGy for a total of 5,000 cGy.

Restaging and MRI

**Figure 4:**
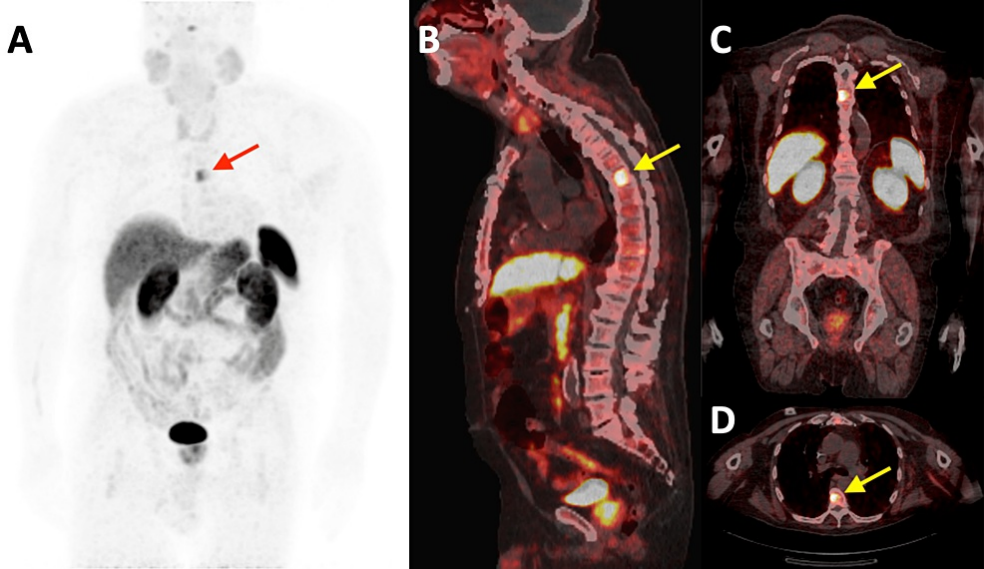
Post-treatment Ga-68-DOTATATE PET-CT study. A. Coronal maximum intensity projection image of the Ga-68-DOTATATE PET study illustrating interval resolution of the increased radiotracer activity in the left upper extremity and axilla when compared to the pre-treatment exam. A new focus of increased radiotracer uptake is seen at the level of the T6 vertebral body (red arrow). B-D. Sagittal (B), coronal (C), and axial (D) fused images of the Ga-68-DOTATATE PET-CT exam demonstrating focally increased radiotracer uptake within the T6 vertebral body (yellow arrows). PET-CT: positron emission tomography-computed tomography

A repeat Ga-68-DOTATATE PET-CT was performed three months following treatment. There was complete interval resolution of the left upper arm and axillary masses with no residual radiotracer uptake in the left upper extremity or axilla. The MIP (Figure 4A), sagittal (Figure 4B), coronal (Figure 4C), and axial (Figure 4D) fused images revealed a new focus of increased radiotracer uptake in the T6 vertebral body, not seen on the pretreatment PET-CT. These findings were suspicious for distant metastasis.

**Figure 5:**
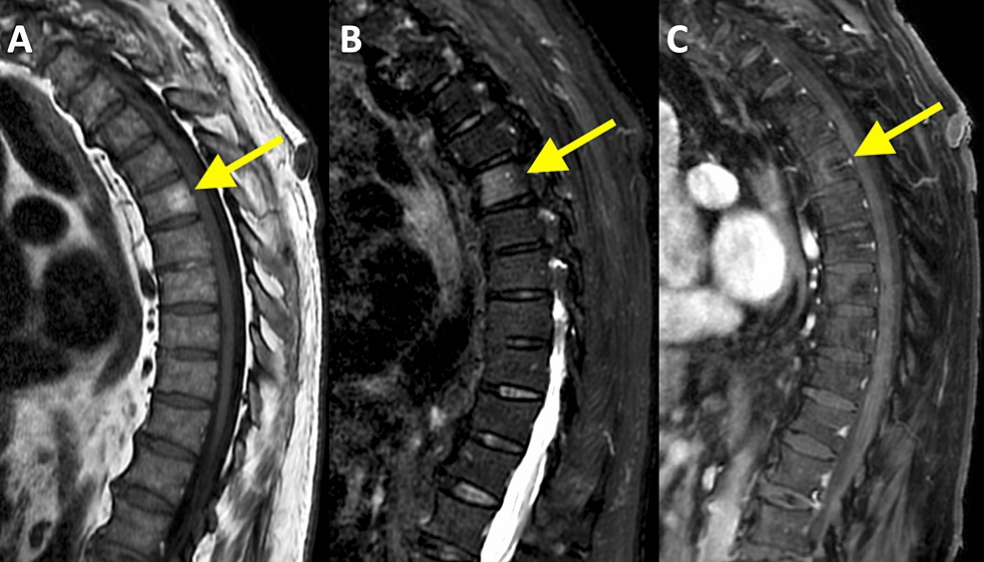
A. Sagittal image of a T1 FLAIR MRI sequence demonstrating a well-circumscribed region of increased T1 signal intensity in the T6 vertebral body (yellow arrow). B. Sagittal image of a STIR MRI sequence illustrating corresponding increased STIR signal intensity in the T6 vertebral body (yellow arrow). C. Sagittal image of a post-contrast T1-weighted MRI sequence with fat suppression revealing non-enhancement of the T1 and T2 hyperintense lesion (yellow arrow). FLAIR: fluid-attenuated inversion recovery; MRI: magnetic resonance imaging; STIR: short tau inversion recovery

A subsequent MRI of the thoracic spine with a gadolinium-based contrast agent was performed. A well-circumscribed lesion in the T6 vertebral body demonstrating hyperintense signal on both sagittal T1-fluid-attenuated inversion recovery (Figure 5A) and short tau inversion recovery (Figure 5B) sequences was identified, corresponding to the Ga-68-DOTATATE-avid lesion. Post-contrast T1-weighted sagittal images with fat suppression (Figure 5C) revealed no enhancement of the lesion. Findings were consistent with a benign vertebral hemangioma.

## Discussion

MCC is a rare, aggressive, and deadly dermatologic malignancy of neuroendocrine origin typically seen in the elderly Caucasian population [1-3]. Although the pathogenesis is not completely understood, immunosuppression and Merkel cell polyomavirus infection have interestingly been implicated in the development of this uncommon disease [4]. The case presented here was seen in a South American male with tan-colored skin and no history of immunosuppression.

MCC is characterized by a painless and rapidly growing erythematous skin nodule, as was described in the patient presented here. Diagnosis is confirmed with biopsy as tissue samples demonstrate intermediate, small-cell, or trabecular subtype histopathological characteristics along with CK20, synaptophysin, and chromogranin positivity [7,8].

Radiologic imaging of MCC is non-specific, demonstrating hypervascular lobulated soft tissue masses in the superficial soft tissues, as seen on the ultrasound and MRI findings presented here. Nuclear imaging, however, serves as a valuable tool for evaluating the extent of disease and disease recurrence. Ga-68-DOTATATE has emerged as a key radiotracer with a reported 97% sensitivity and 95.1% specificity for neuroendocrine tumors owing to its properties as a somatostatin receptor analog [5]. In fact, Epstude et al. demonstrated increased sensitivity of Ga-68-DOTATATE for metastatic MCC lesions when compared to FDG on PET-CT [9].

In this patient, a new focus of increased radiotracer uptake was identified in the T6 vertebral body on the post-treatment restaging Ga-68-DOTATATE PET-CT, raising concern for metastasis. However, subsequent contrast-enhanced MRI revealed a well-circumscribed, non-enhancing intraosseous lesion corresponding to the suspicious focus with both intrinsic T1 and T2 hyperintense signal. These findings are most consistent with benign vertebral hemangiomas which, although classically enhance following contrast administration, may show variable enhancement patterns [10].

Multiple benign spinal pathologies such as hemangiomas, meningiomas, and Schmorl’s nodes have been reported to demonstrate increased Ga-68-DOTATATE uptake [5,11,12]. In fact, a study by Skoura et al. reviewed 1,258 Ga-68-DOTATATE scans and demonstrated a false-positive rate of 1.11% and a false-negative rate of 2.3% [5]. The majority of the false-positive cases were due to uptake in normal somatostatin receptor-expressing organs (such as the pancreas and adrenal gland) or due to inflammatory changes. The percentage of benign vertebral hemangiomas that exhibit increased radiotracer uptake is unknown. In our patient, the increased radiotracer activity at T6 was likely obscured on the initial pre-treatment PET-CT due to the much more somatostatin-avid lesions in the left upper extremity and axilla. Following treatment, however, the vertebral hemangioma was uncovered.

These findings are an important pitfall of the use of Ga-68-DOTATATE in the staging of MCC, as increased radiotracer uptake on PET imaging can raise concern for metastatic disease. Further characterization of such foci in the spine should be performed with contrast-enhanced MRI. Reassuring imaging findings suggesting a benign hemangioma include intrinsic hyperintense T1 and T2 signal, well-circumscribed margins, absence of extraosseous extension, and minimal to no enhancement on post-contrast sequences [10]. Recent literature has described Ga-68-pentixiafor as a novel radiopharmaceutical agent with greater sensitivity in identifying MCC lesions on PET-CT when compared to Ga-68-DOTATATE [6]. However, further research is needed to accurately judge its use as a reliable radiotracer for this rare entity.

## Conclusions

MCC is a highly aggressive neuroendocrine malignancy of the dermis which typically presents as a rapidly enlarging erythematous nodule. Diagnosis is made with physical examination and biopsy of the skin lesion with histopathological analysis. Radiologic imaging can also be performed, primarily serving as a tool in the staging and management of this rare entity.

Cancer staging and evaluation for recurrence can be performed with Ga-68-DOTATATE, a somatostatin receptor analog that demonstrates high sensitivity and specificity for neuroendocrine tumors. The case described here illustrates the utility of Ga-68-DOTATATE in staging MCC, as well as an important pitfall for clinicians to consider in the setting of benign vertebral hemangiomas which can mimic metastasis.
